# Molecular Basis for the Selectivity of DHA and EPA in Sudlow’s Drug Binding Sites in Human Serum Albumin with the Combined Use of NMR and Docking Calculations

**DOI:** 10.3390/molecules28093724

**Published:** 2023-04-26

**Authors:** Eleni Alexandri, Themistoklis Venianakis, Alexandra Primikyri, Georgios Papamokos, Ioannis P. Gerothanassis

**Affiliations:** Section of Organic Chemistry and Biochemistry, Department of Chemistry, University of Ioannina, GR-45110 Ioannina, Greece

**Keywords:** HSA, DHA, EPA, STD NMR, INPHARMA NMR, docking calculations

## Abstract

Medium- and long-chain saturated and unsaturated free fatty acids (FFAs) are known to bind to human serum albumin (HSA), the main plasma carrier protein. Atomic-level structural data regarding the binding mode in Sudlow’s sites I (FA7) and II (FA4, FA3) of the polyunsaturated ω-3 fatty acids docosahexaenoic acid (DHA) and eicosapentaenoic acid (EPA), however, are largely unknown. Herein, we report the combined use of saturation transfer difference (STD) and Interligand NOEs for Pharmacophore Mapping (INPHARMA) NMR techniques and molecular docking calculations to investigate the binding mode of DHA and EPA in Sudlow’s sites Ι and ΙΙ of HSA. The docking calculations and the significant number of interligand NOEs between DHA and EPA and the drugs warfarin and ibuprofen, which are stereotypical ligands for Sudlow’s sites I and II, respectively, were interpreted in terms of competitive binding modes and the presence of two orientations of DHA and EPA at the binding sites FA7 and FA4. The exceptional flexibility of the long-chain DHA and EPA and the formation of strongly folded structural motives are the key properties of HSA–PUFA complexes.

## 1. Introduction

**Figure 1:**
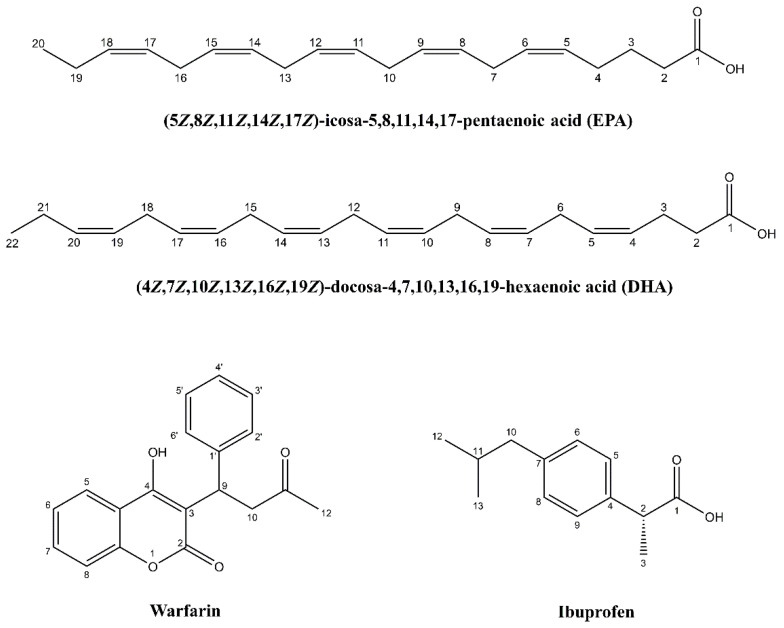
Molecular structures of EPA, DHA, warfarin, and ibuprofen with the numbering of atoms.

Polyunsaturated omega-3 (ω-3) free fatty acids (FFAs), such as docosahexaenoic acid (DHA) and eicosapentaenoic acid (EPA) (Figure 1), are known to have important roles in human health and disease [1,2,3,4]. DHA and EPA are components of membrane glycerophospholipids (GPLs); they bind to several receptor proteins and act as precursors of lipid mediators [5,6]. DHA, for example, is a cell membrane component that plays an important role in optimal brain development and function [3] and binds to the retinoid X-receptor in the mouse brain [7] and the human brain FA-binding protein [8,9]. Recently, linolenic acid and EPA were shown to significantly block the entry of SARS-CoV-2 [10].

Human serum albumin (HSA) is an abundant and versatile protein in plasma and interstitial fluids that maintains oncotic pressure, interacts with a number of cell surface receptors, transports thyroid hormones and hormones that are fat-soluble, and carries a variety of free fatty acids to the liver and to myocytes for utilization of energy [11,12,13,14,15]. HSA mediates the binding of and transports a variety of drugs, amino acids, and nutrients. HSA is a monomeric protein composed of 585 amino acids (MW~66 kDa). X-ray structure determination has shown that HSA is a globular, heart-shaped protein composed mainly of α-helices (~67%) without β-sheets [16,17]. It is arranged in three separate functional domains (I, II, and III), which are subdivided into A and B subdomains: IA (1–110 a.a.), IB (113–195 a.a.), IIA (196–303 a.a.), IIB (304–383 a.a.), IIIA (384–500 a.a.), and IIIB (501–585 a.a.). The domains vary in terms of the numbers and composition of non-polar and polar amino acid residues.

The various fatty acid and drug-binding sites have been investigated extensively over the past 40 years [12,13,14,15,18,19,20]. X-ray crystallographic [16,17,18,21,22,23,24] and NMR [24,25,26,27] studies revealed the presence of seven to nine binding sites that may be occupied by both medium- and long-chain saturated fatty acids, with chain lengths from C10 to C18. FFA binding sites are asymmetrically distributed throughout the protein. Binding studies have indicated that saturated and monounsaturated fatty acids interact similarly with serum albumin; polyunsaturated free fatty acids (PUFAs), however, may show rather distinct binding behavior [20]. Furthermore, in vivo studies have demonstrated that two thirds of the FFAs bound to serum albumin are unsaturated under normal conditions.

Two primary sites of interactions between serum albumin and drugs have been revealed [16,17,18]. Sudlow’s site I (FA7) mediates the binding and transport of bulky heterocyclic anions, such as warfarin, phenylbutazone, azapropazone, indomechacin, and several other therapeutic compounds. Sudlow’s site II (FA4 and FA3), which is highly conserved, mediates the binding and transport of aromatic carboxylates, such as ibuprofen, digitoxin, benzodiazepine, propofol, and non-steroid anti-inflammatory drugs, in an extended conformation.

Competition between free fatty acids and drugs for Sudlow’s sites I and II may increase the free fraction of the drugs, thereby affecting potency and their pharmacokinetic behavior. Furthermore, although the serum albumin binding assay is the usual procedure for the development of new drugs, the binding modes of the polyunsaturated FFAs in Sudlow’s sites I and II have not been elucidated. We report herein structural aspects of the binding modes of the polyunsaturated free fatty acids DHA and EPA to HSA with the combined use of saturation transfer difference (STD) [28,29,30], Tr-NOESY [31,32,33], and Interligand NOEs for Pharmacophore Mapping (INPHARMA) [26,27,34,35,36] NMR techniques and molecular calculations [27,34,35,36,37,38,39,40]. Particular emphasis was given to competition experiments with warfarin and ibuprofen, which are stereotypical ligands for Sudlow’s site I (FA7) and II (FA4, FA3) of HSA [16,17,18,19]. Since ibuprofen shows weak binding to FA6, computational calculations were also performed for this binding site.

## 2. Results and Discussion

### 2.1. STD and INPHARMA NMR Competition Experiments on DHA and EPA with Warfarin and Ibuprofen

#### 2.1.1. The Binding Site FA7

**Figure 2:**
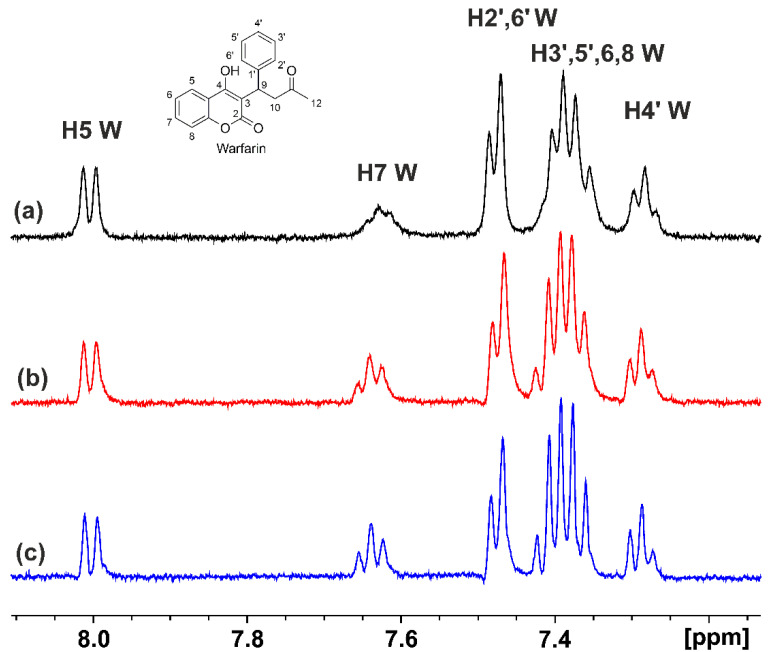
Selected regions of the STD NMR spectra: (**a**) of warfarin (W) (1.25 mM) with HSA (25 μΜ) in 50 mM PBS buffer in D_2_O with 10% DMSO-*d*_6_; (**b**) after the addition in solution (**a**) of DHA (2.5 mM); (**c**) after the addition in solution (**a**) of EPA (2.5 mM) (T = 310 K, number of scans = 80, saturation time = 2 s, experimental time = 52 min for (**a**–**c**).

Warfarin is an anti-coagulant drug that shares this binding site, designated as site I in subdomain IIA, with a wide range of drugs, such as phenylbutazone, tolbutamide, and indomethacine [16,17,18,19]. Various complexation studies on warfarin with HSA/BSA have been reported, showing a wide range of formation constants (10^4^–10^5^ M^−1^) depending on the experimental conditions used [41]. The STD NMR spectrum of the aromatic region of warfarin with HSA is shown in Figure 2a. The addition of DHA (at mole ratios of DHA/warfarin ≈ 1:1 and 2:1) reduced the linewidths of the STD signals of the aromatic protons H7, H3′, 5′, 6, 8, and H4′ without any significant difference in the STD intensity (Figure 2b). The addition of EPA (at mole ratios of EPA/warfarin ≈ 1:1 and ≈2:1) resulted in reductions in the linewidths of the STD signals of all the aromatic resonances of warfarin, most notably those of H7, H2′, 6′, H3′, 5′, 6, 8, and H4′, without a significant difference in the STD intensity (Figure 2c). This can be attributed to enhanced binding of warfarin to secondary binding sites [16]. It has been shown that addition of up to three moles of long-chain FFAs enhances the binding of ligands in Sudlow’s site I (FA7), presumably due to the expansion of the size of the cavity [16,17,18]. Furthermore, for several drugs, secondary binding sites outside subdomain IIA were observed in the HSA–myristate complex [17]. Interestingly, weak secondary binding of warfarin in subdomain IB was also observed in the absence of fatty acid. Nevertheless, to assess whether the reduction in the linewidths of the STD signals reflected competitive interactions in relation to binding site FA7, rather than allosteric phenomena, the INPHARMA NMR technique was applied [26,27,34,35,36,42]. This approach relies on the inter-NOE connectivities of two ligands, provided that they share a common binding site with a distance < 5 Å. The numbers and magnitudes of such intermolecular NOEs can be utilized to derive the relative orientation of the two ligands at a common binding site.

**Figure 3:**
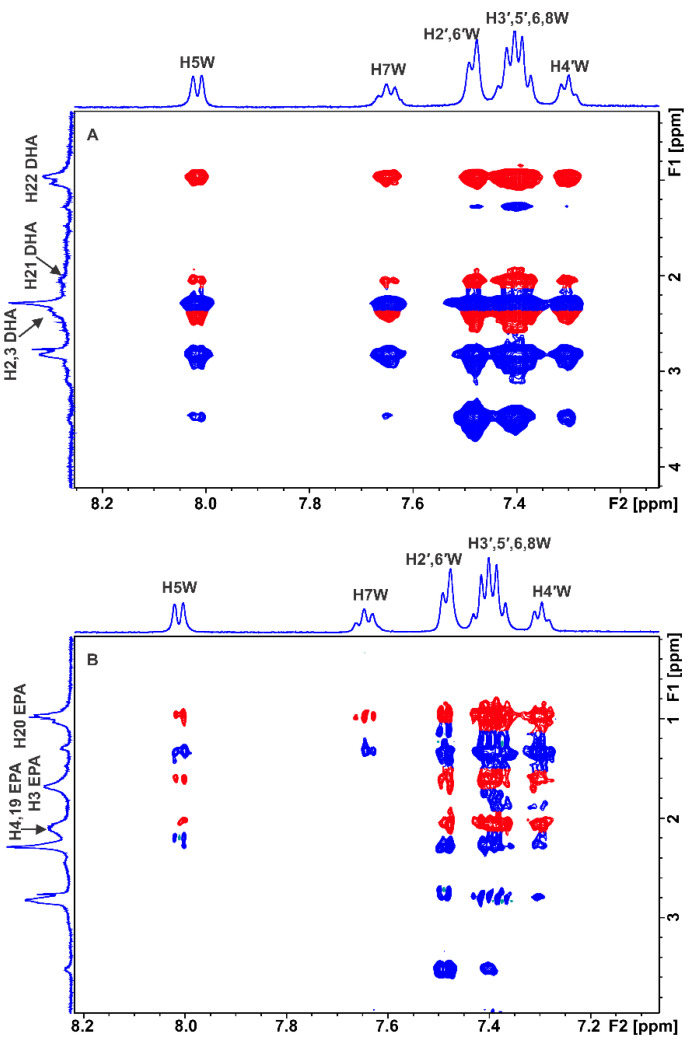
Selected regions of interligand 2D Tr-NOESY NMR spectra for warfarin (W) (2.5 mM) with HSA (25 μΜ) in 50 mM PBS buffer in D_2_O with 10% DMSO-*d*_6_ after the addition of DHA (2.5 mM) (**A**) and EPA (2.5 mM) (**B**) (mixing time = 300 ms, number of scans = 112, experimental time = 17 h). The red cross-peaks denote interligand NOE connectivities. The blue cross-peaks denote intraligand NOE connectivities of warfarin (W).

Figure 3 shows a significant number of interligand NOEs shared between DHA and EPA and warfarin (red cross-peaks), demonstrating that they share a common binding site at FA7. Of particular interest are the common inter-NOEs shared between the H2,3 and terminal CH_3_ groups of DHA/EPA and the aromatic protons of both the benzopyran and the phenyl butyl rings of warfarin, although the crystallographic distance of the two aromatic systems is ~6.9 Å [16,17,18,19,20,21,22,23,24,25,26,27]. These results can be attributed to two anchor binding sites within the FA7 binding pocket (see the discussion on computations).

Sledz et al. [43] investigated interligand NOEs of highly hydrophobic inhibitors of *Mycobacterium tuberculosis* pantothenate synthetase (PtS). Negative interligand NOEs were observed even in the case of perdeuterated proteins. This was attributed to the formation of high-molecular-weight aggregates of the inhibitors in aqueous environments, which results in negative NOEs that are not mediated through specific interactions with the protein. Since FFAs in an aqueous solution show the presence of negative NOEs due to the formation of micellar aggregates [27], it was necessary to perform further 2D Tr NOESY experiments to investigate whether the drug warfarin is incorporated within the micellar system. Positive intra-NOEs (anti-phase with respect to the diagonal) were observed for warfarin between H2′, 6′ and H9 and H10 due to their spatial proximity (Figure S1, Supplementary Materials), without any cross-peaks with DHA. Similar results were obtained with the binary mixture of EPA with warfarin (Figure S2, Supplementary Materials). This demonstrates that warfarin is not incorporated within the micellar system, and its correlation time, τ_c_, is within the extreme narrowing condition (ω_0_ τ_c_ << 1). Therefore, the interligand NOEs shown in Figure 3 did not originate from the direct transfer of magnetization between DHA/EPA and warfarin since cross-peaks were not observed in a control spectrum acquired for a solution containing only binary mixtures of ligands in the absence of HSA.

#### 2.1.2. The Binding Site FA4

**Figure 4:**
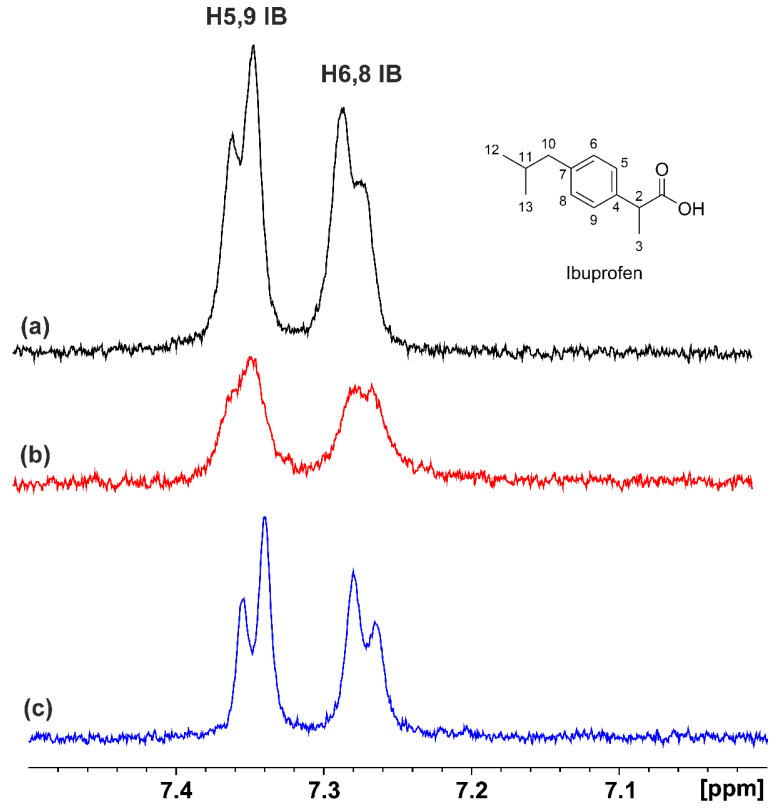
Selected regions of the STD NMR spectra: (**a**) of ibuprofen (IB) (2.5 mM) with HSA (25 μΜ) in 50 mM PBS buffer in D_2_O with 10% DMSO-*d*_6_; (**b**) after the addition in solution (**a**) of DHA (2.5 mM); (**c**) after the addition in solution (**a**) of EPA (2.5 mM) (T = 310 K, number of scans = 80, saturation time = 2 s, experimental time = 52 min for (**a**–**c**).

**Figure 5:**
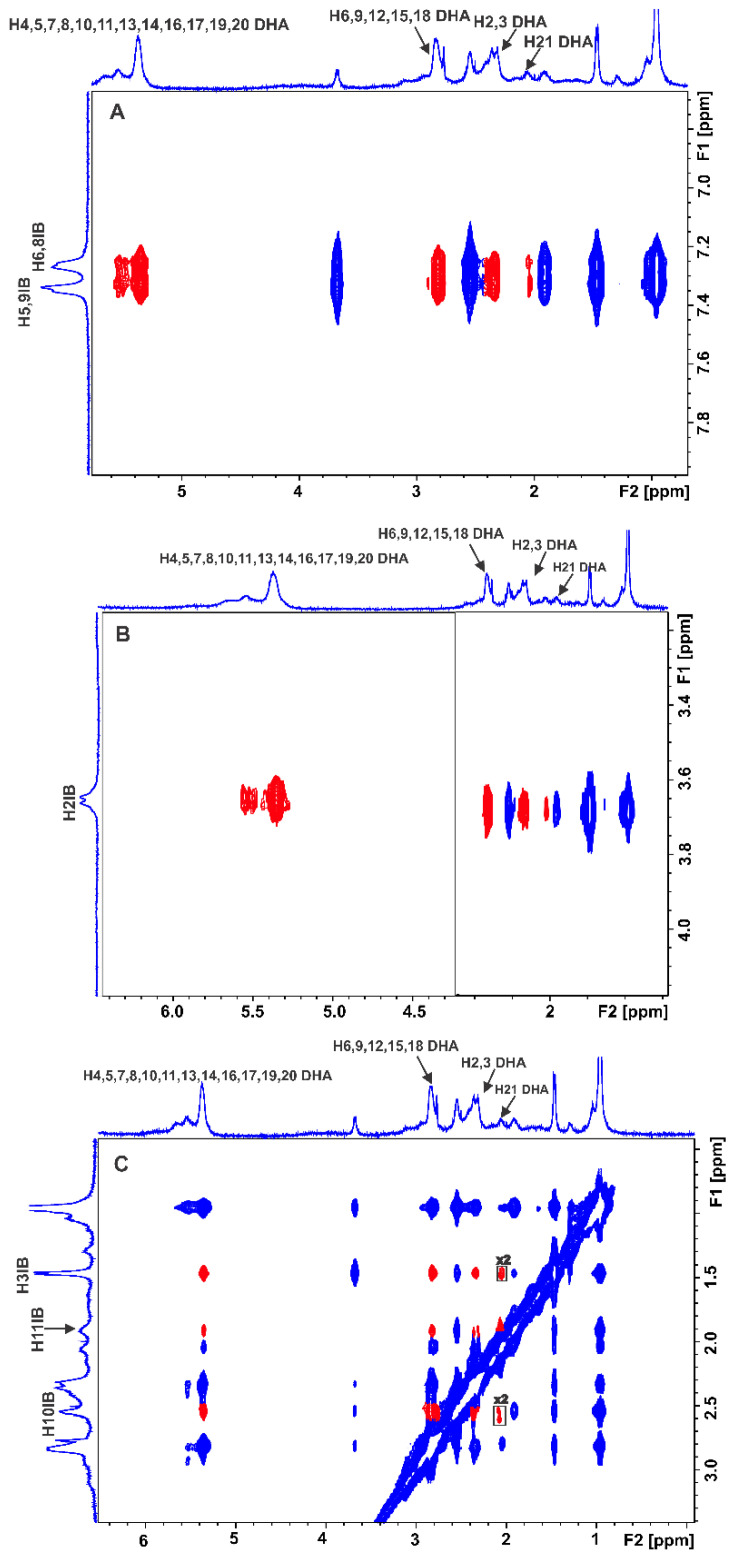
Selected regions of interligand 2D Tr-NOESY NMR spectra for DHA (2.5 mM) with HSA (25 μΜ) in 50 mM PBS buffer in D_2_O with 10% DMSO-*d*_6_ after the addition of ibuprofen (IB) (2.5 mM) (mixing time = 300 ms, number of scans = 112, experimental time = 17 h). The red cross-peaks denote the interligand NOE connectivities of H5,9, H6,8 (**A**), H2 (**B**), and H10,11,3 (**C**) of ibuprofen with DHA. The blue cross-peaks denote the intraligand NOE connectivities of ibuprofen (IB) and DHA.

Similar STD competition experiments as those for the warfarin FA7 binding site were also performed with ibuprofen (a non-steroidal anti-inflammatory drug), which shows a high affinity for albumin, with binding constants up to 6 × 10^6^ M^−1^ [44]. The STD NMR spectrum of ibuprofen in complexation with HSA is shown in Figure 4. The addition of DHA (at a mole ratio of DHA/ibuprofen ≈ 1:1) and EPA (at a mole ratio of EPA/ibuprofen ≈ 1:1) resulted in significant reductions in the intensities of the STD signals of the aromatic protons of ibuprofen by factors of ~43% and ~17%, respectively. The binding of the exogenous drug ibuprofen probably reflected competition with DHA and EPA for the same FA4 binding site of HSA. Again, to assess whether the reduction in the STD signals reflected competitive interactions rather than allosteric phenomena, the INPHARMA NMR technique was applied. Figure 5 and Figure S3 (Supplementary Materials) show significant interligand NOEs shared between DHA/EPA and ibuprofen (red cross-peaks). Since FFAs in aqueous solution show the presence of negative NOEs due to the formation of micellar aggregates, it was necessary to perform further 2D Tr NOESY experiments to investigate whether the drug ibuprofen was incorporated within the micellar system. Positive intra-NOEs were observed without cross-peaks for ibuprofen with DHA/EPA (Figure S4 (Supplementary Materials)). This demonstrates that ibuprofen was not incorporated within the micellar system, and its correlation time, τ_c_, was within the extreme narrowing condition (ω_0_ τ_c_ << 1). The interligand NOEs, therefore, in Figure 5 and Figure S3 (Supplementary Materials) did not originate from the direct transfer of magnetization between DHA/EPA and ibuprofen since cross-peaks were not observed in a control spectrum acquired for a solution containing only binary mixtures of ligands in the absence of HSA.

Common interligand NOEs were observed between H2 and H3 of ibuprofen and the H2,3 of DHA and H2, H3 of EPA. This indicated a common binding mode for the carboxylate group of ibuprofen and those of DHA/EPA, which was in agreement with literature data on the primary role of electrostatic interactions [24,25]. Of particular interest are the interligand NOEs shared between H11, H10 of ibuprofen and H2,3 and H21 of DHA and H2, H3 of EPA. However, the proton distances for a single anchor binding site were beyond the detection limits of NOEs. This can be attributed to the presence of two anchor binding sites, as suggested by the X-ray structure determination [23] and the combined use of NMR and docking calculations [27,42] (see the discussion on computations).

### 2.2. Docking Calculations

#### 2.2.1. The Binding Site FA7

The X-ray structure determination of the binding site FA7 (subdomain IIA) showed the presence of two clusters of polar residues: Tyr-150, His-242, and Arg-257 (inner cluster) and Lys-195, Lys-199, Arg-218, and Arg-222 (external cluster at the entrance of the pocket). The benzoyl moiety of warfarin forms hydrophobic interactions with Arg-218 and the side chain of Tyr-214. The O4 atom of warfarin forms hydrogen bonds with Nε_2_ of His-242 (2.9 Å), the O2 with Nε of Arg-222, and the acetyl oxygen with NH_2_ of Arg-222 [16]. In contrast, crystallographic analysis of medium- (C10:0, C12:0, C14:0, C16:0) and long-chain (C18:0, C18:1, and C20:4) free fatty acids showed no clearly defined coordination of the FA carboxylic groups, which was attributed to the low affinity of FFAs for the FA7 binding site [22,23].

**Figure 6:**
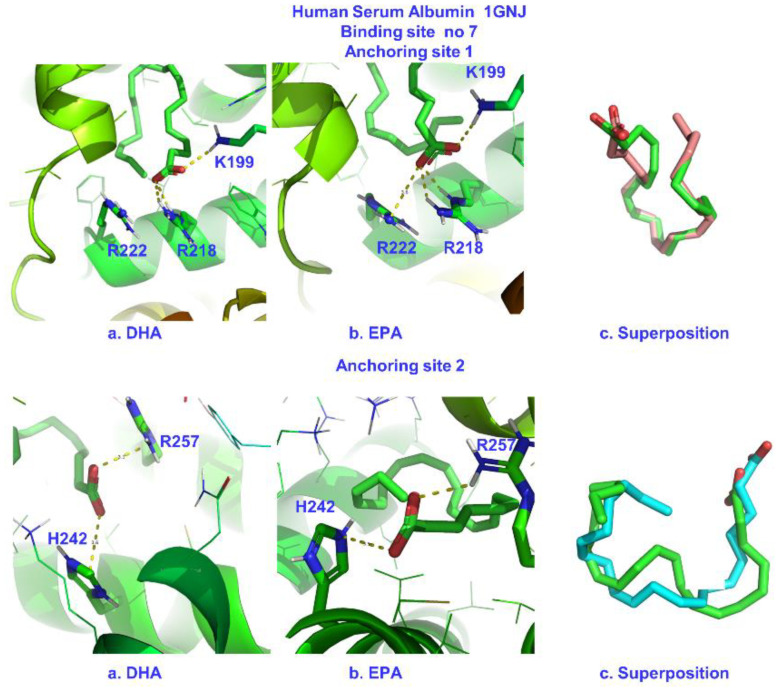
Poses for binding site FA7 of HSA with DHA (**a**) and EPA (**b**) for the two anchoring sites (up and down) and their superpositions (**c**).

**Figure 7:**
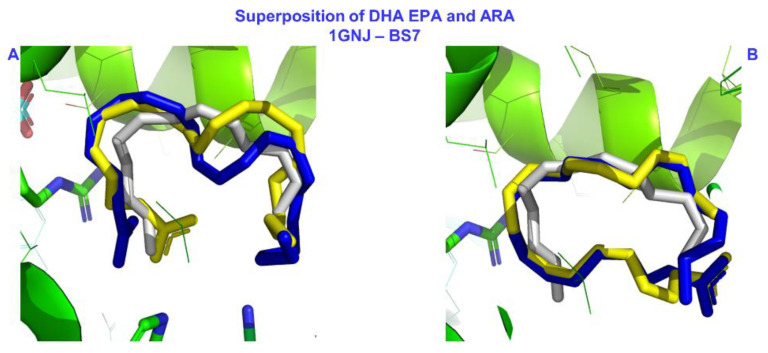
The superposition of poses one and two for DHA and EPA in panels (**A**) and (**B**), respectively, generated via molecular docking in the current work and the crystallographic structure of ARA bound in FA7 of HSA (PDB code: 1GNJ.pdb) [23]. Color code: DHA—blue, EPA—yellow, ARA—gray. In the crystallographic structure of ARA, the carboxylate group is absent due to low density.

The results of the docking calculations and the poses that describe the interactions of the two FFAs (DHA and EPA) with HSA at the binding site FA7 are given in Table 1 and Figure 6 and Figure 7. The identified interactions between the protein and the ligands show a clear anchoring preference for the amino acids we identified in our previous work [27]. The first two poses from our results for DHA showed that the amino acids forming anchoring site one (Lys-199, Arg-218, Arg-222), also identified by X-ray crystallography [22], achieved a high binding score (−7.0 kcal/mol) via electrostatic interactions with the carboxylate group. The second anchoring site (His-242 and Arg-257) was also identical to the one we described in previous work [27], with the same binding score (−7.0 kcal/mol). The distances that defined the binding in the two anchoring sites between the FFA and HSA were typical for electrostatic interactions between oppositely charged residues and their interfering groups (3.0–3.6 Å in Table 1). The same anchoring sites with the standard distances of an electrostatic interaction were identified for EPA in poses two and seven, respectively. Our results also showed that DHA and EPA bound to HSA in FA7 via a folded structure, a common geometry for these FFAs in the liquid state [45,46]. Based on crystallographic ambiguity, FA7 was considered a lower affinity site for FFAs and a preferred spot for shorter FAs [23]. Our present and previous [27] results reveal two binding configurations (in the two anchor sites, respectively) at the same site. This explanation was further supported by the inspection of the superposition of our poses for DHA and EPA and the crystal structure of arachidonic acid (ARA) (IUPAC name: (5*Z*,8*Z*,11*Z*,14*Z*)-icosa-5,8,11,14-tetraenoic acid)—the HSA complex in FA7, as shown in Figure 7. Arachidonic acid has one and two fewer allylic double bonds than EPA and DHA, respectively, and it is the only polyunsaturated fatty acid with a known X-ray structure determination [23]. Nevertheless, the crystal structure of ARA does not include the carboxylate group (see Figure 3f in [23]), and our poses adopted similar spatial arrangements with ARA for both anchoring sites. The lack of electron density for the carboxylate group of ARA in the X-ray structure can be attributed to the rapid interconversion of ARA among the two anchoring groups in the FA7 binding pocket.

Binding affinity depends on the number of bis-allylic double bonds; both EPA and DHA possess a higher affinity with respect to caproleic, oleic, and linoleic acids, and it is only slightly weaker than that of warfarin (W) [27]. Polyunsaturated free fatty acids rapidly convert between a large number of conformers with structural motives such as spiral or hairpin motives in the liquid and solution states [45,46,47]. These pre-existing conformations, combined with the specific charge and hydrogen bond interactions, as well as π-interactions, of the polyunsaturated chains, may be the key properties for HSA–PUFA interactions in the FA7 binding site.

#### 2.2.2. The Binding Site FA4

The binding site FA4 has been characterized by X-ray crystallography as a long and narrow hydrophobic tunnel [22,23]. The carboxylate group of ibuprofen makes salt bridges with Lys-414 and Arg-410 (PDBid: 2BXG). The X-ray electron densities for the C18:0, C18:1, and C20:4 fatty acid ligands showed broadened features at each end of the pocket, suggesting that fatty acids are capable of binding in two orientations within the pocket, with the carboxylate groups being hydrogen-bonded with Arg-410, Tyr-411, and Ser-489 (anchor site one) [22,23]. This site is also a dual site: a second anchoring site has been identified composed of Ser-419 and Thr-422 (anchor site two) that hosts fatty acids with long chains [22,23]. In the case of the polyunsaturated ARA (C20:4), a dominant configuration has been suggested [22,23] with salt bridges to Arg-410, Tyr-111, and Ser-489.

**Figure 8:**
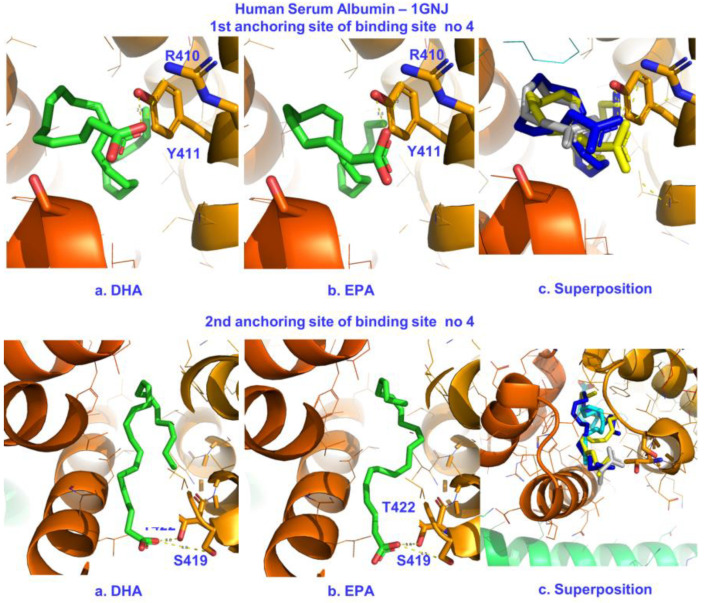
Poses of the electrostatic and hydrogen bond interactions between the carboxylate groups of DHA/EPA (**a**,**b**) and Arg-410/Tyr-411 (up) and Ser-419/Tyr-422 (down) in the binding site FA4 of HSA. The superposition of molecular docking poses and the crystallographic structure of ARA in the same binding site of HSA [23] are shown in (**c**). Color code for (**c**): DHA—blue, EPA—yellow, ARA—gray.

Our docking results for FA4 are shown in Table 2 and Figure 8. The affinities of DHA and EPA for anchor site one were −7.5 and −7.0 kcal/mol, comparable with the binding affinity of ibuprofen (−7.3 kcal/mol). We identified a pose where the carboxylate groups of DHA and EPA adopted the second orientation and located interactions with Ser-419 and Thr-422. For anchor site two, the binding affinities were even higher for both FFAs (−7.8 kcal/mol). The conformations of DHA and EPA preferred a folded geometry in both anchoring sites (Figure 8) in contrast to ARA.

#### 2.2.3. The Binding Site FA3

**Figure 9:**
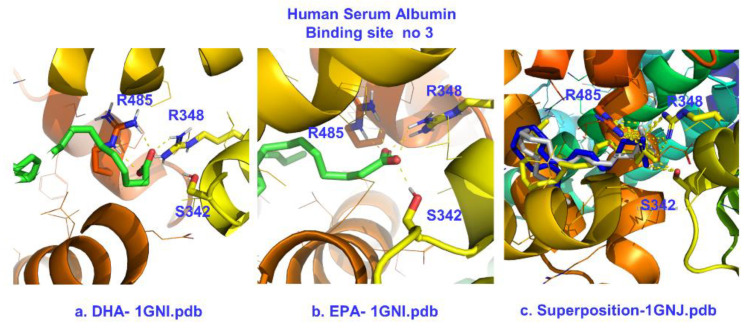
Poses with best scores for the binding site FA3 of HSA with DHA (**a**) and EPA (**b**), superposition of molecular docking poses, and crystallographic structure of ARA bound in FA3 of HSA (**c**) [22,23].

X-ray structural data for the C10:0, C12:0, C14:0, C16:0 C18:0, and C20:4 FFAs in the binding site FA3 (subdomains IIIA and IIB) showed identical anchoring behavior, with strong electrostatic and hydrogen bond interactions with Ser-342, Arg-348, and Arg-485 [22,23]. The findings of our molecular docking calculations regarding binding site FA3 are presented in Table 3 and Figure 9. This site generates higher affinities of −8.3 and −7.9 kcal/mol for DHA and EPA, respectively (Table 3). These values were the highest calculated for all sites and even higher than ibuprofen (−7.2 kcal/mol) [27]. This was in excellent agreement with the strong reductions in the STD NMR intensities of ibuprofen upon the addition of DHA/EPA (Figure 4). The characteristic residues Ser-342, Arg-348, and Arg-485 (Figure 9) generated strong electrostatic and hydrogen bond interactions with the carboxylate group. The spatial orientation adopted by DHA and EPA was almost identical to ARA (Figure 9c). The FA3 pocket can host a maximum of 12–14 methylene units and imposes unfavorable U-bends on long fatty acids. However, such U-bends may favor PUFAs since folding becomes an intrinsic property of FFAs with four and five bis-allylic double bonds. In recent work, the ability of DHA and EPA to adopt folded conformations was highlighted [45,46].

Our findings, corroborated by the scientific literature, suggest that FA3 may become a high-affinity pocket for long-chain polyunsaturated free fatty acids since their folded conformation matches the spatial arrangement demands of the site. The folding of PUFAs may be a functional prerequisite since a theoretical study has linked the diameters of DHA helices (~4.7 Å) with the opening of hydrophobic “channels” within the membranes [47].

#### 2.2.4. The Binding Site FA6

**Figure 10:**
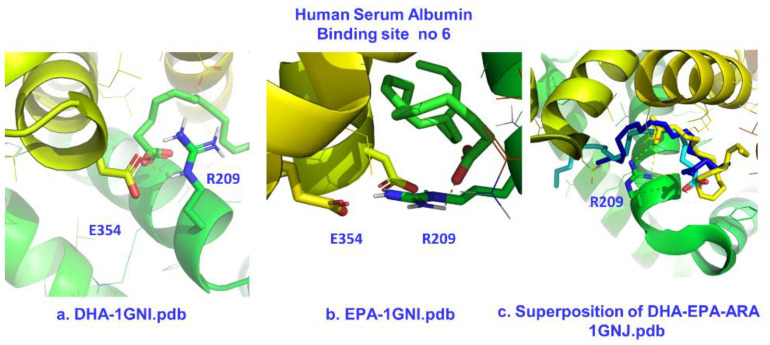
Poses of the electrostatic interactions between the carboxylate groups of DHA and EPA and Arg-209 in the binding site FA6 of HSA (**a**,**b**) and superposition of molecular docking poses and the crystallographic structure of ARA for the same site. For (**a**,**b**), the crystal structure with the PDB code 1GNI.pdb was employed [23]. The superposition was generated based on the crystal structure of HSA with ARA with the PDB code 1GNJ.pdb. Color code for (**c**): DHA—blue, EPA—yellow, ARA—gray.

Our molecular docking results for DHA and EPA in binding site FA 6 (subdomains IIA and IIB) also showed higher affinities of −7.0 and 6.9 kcal/mol (Table 4) with respect to FFAs with smaller numbers of double bonds [27]. The primary electrostatic interaction is between Arg-209 and the carboxylate group (Figure 10). Nevertheless, this interaction may not be of maximum strength. Arg-209 is already involved in salt-bridge formation with Glu-354 (Figure 10a,b) and Asp-324, as observed in the crystal structure (PDB code: 1GNJ.pdb) [23]. Glu-354 also interacts with Lys-351. These endogenous interactions may explain why the carboxylate groups of various free fatty acids lacked conserved electrostatic interactions in the X-ray structure determination. The calculated high affinities of −7.0 and −6.9 kcal/mol may have resulted from possible favorable interactions between the longer polyunsaturated fatty acid (PUFA) chain of four and five bis-allylic double bonds. This result is comparable to the binding affinity of ibuprofen for the same site (−6.8 kcal/mol) [27].

## 3. Materials

EPA, purity > 99%, and DHA, purity > 99%, were purchased from Larodan AB, Solna, Sweden. Human serum albumin fatty acid-depleted lyophilized powder, purity ≥ 96% (agarose gel electrophoresis), and warfarin and ibuprofen, purity ≥ 98% (GC), were purchased from Sigma Aldrich Chemie GmbH, Taufkirchen, Germany.

## 4. NMR Experimental Procedures

STD NMR experiments were performed at 37 °C on Bruker AV-500 and Bruker AV-NEO-500 spectrometers (Bruker Biospin, Rheinstetten, Germany) in the presence of HSA (25 μM) and 50 mM PBS (pD 7.4) in D_2_O with 10% DMSO-*d*_6_. To facilitate the dissolution of DHA and EPA, they were first dissolved in DMSO-*d*_6_ and then diluted in PBS. The solution was heated at 50 °C for 5 min before the addition of HSA. The selective drugs warfarin and ibuprofen were added in the solution for the competition experiments in the concentrations specified in the figure captions. Selective saturation was achieved for 2 s through the use of a train of Gaussian-shaped pulses, as previously reported [26,27]. The excitation profile of the Gaussian-shaped pulse was verified to be <0.8 ppm; therefore, the on-resonance excitation pulse was set to at least 1 ppm from the nearest ligand resonances. An excitation sculpting pulse sequence was used for water suppression.

Tr-NOESY and INPHARMA NMR experiments were carried out at 37 °C in the presence of I (25 μM) in 50 mM PBS (pD 7.4) in D_2_O with 10% DMSO-*d*_6._ The concentrations of DHA, EPA, warfarin, and ibuprofen are specified in the figure captions. Mixing times of 100, 200, and 300 ms were used to verify the linearity region of the Tr-NOE intensity. Solvent suppression was achieved using an excitation sculpting scheme.

## 5. Computational Methods

The following human serum albumin (HSA) structures were obtained from the Protein Data Bank: 1GNI (HSA complexed with oleic acid) and 1GNJ [23]. The structures of EPA (IUPAC name: (5*Z*,8*Z*,11*Z*,14*Z*,17*Z*)-eicosa-5,8,11,14,17-pentaenoic acid), DHA (IUPAC name: (4*Z*,7*Z*,10*Z*,13*Z*,16*Z*,19*Z*)-docosa-4,7,10,13,16,19-hexaenoic acid), ibuprofen, and warfarin were built with Gauss View 6.0.16 [48]. The deprotonated charged state was adopted for each ligand. The computational approach followed here has been described in our previous work [27]. The docking runs consisted of ten independent runs for each complex. The docking conformations were selected based on the binding affinity for each run. In order to minimize possible irreproducibility issues in the docking methodology [49], the computational results were evaluated and selected based on: (i) the highest affinity for each of ten independent runs, (ii) the minor deviations from existing X-ray crystal structures of unsaturated FFAs with HSA, and (iii) inter-residue NOE intensities resulting from competition INPHARMA NMR experiments. For the molecular docking calculations, the AutoDock Vina1.2 [39] software package was employed. AutoDock Tools 1.5.6 [38] was used as a preprocessing software package to add hydrogen atoms to the protein and select the search space for each complex studied.

## 6. Conclusions

The structural and conformational analyses of DHA and EPA in the Sudlow’s sites of HSA in the present work clearly demonstrate that:

(a) The INPHARMA technique demonstrated the advantage, compared to STD and its variants, of showing numerous strong interligand NOE connectivities between warfarin and DHA/EPA, indicating that both ligands share a common FA7 binding site with distances <5 Å and, thus, are competitive rather than allosteric. The interligand NOEs and docking calculations showed the presence of two conformational states of DHA/EPA due to the presence of two anchoring polar groups of amino acids. DHA and EPA adopt highly folded conformations in the polyunsaturated moiety. Therefore, the exceptional flexibility of the polyunsaturated chain of DHA and EPA is the key property for interactions within the FA7 binding pocket;
(b) In the binding site FA4, the interligand NOEs and docking calculations demonstrated the presence of two anchor sites, in agreement with the FFAs of up to C-18 atoms;
(c) DHA and EPA possess higher FA3 and FA4 affinities with respect to FFAs with shorter chains and PUFAs with smaller numbers of double bonds. The latter finding is entirely consistent with experimental results obtained by employing the fluorescent probe ADIFAB [20];
(d) The approach employing site-specific molecular docking driven by X-ray crystallography and NMR measurements was highly successful in describing the interactions of PUFAs with long chains, since it could minimize known irreproducibility problems in biomedical research [49];
(e) The HSA protein has the ability, through its amino acid sequence, to adopt a multidomain tertiary structure and generate corresponding interactions depending on the size of the molecule. The above property of the protein makes it capable of capturing a wide variety of molecules and playing a pivotal role in health and disease as a carrier of drugs, nutrients, or toxic molecules [12,13,14,15,16,17,18,19].

Further NMR and computational studies are currently in progress to investigate the interactions of synthetic analogues of the free fatty acids [12,50] and primary and secondary oxidations products of FFAs [51,52,53] with HSA.

## Figures and Tables

**Table 1:** Electrostatic and hydrogen bond interactions between the carboxylate groups of DHA and EPA and the amino acids of the binding site FA7 of HSA, the poses that give rise to the interactions, and affinities in kcal/mol.

Ligand	K199			R218			R222			H242			R257		
	Anchor Site One									Anchor Site Two					
	Group	Dist. (Å)	Pose/Affinity (kcal/mol)	Group	Dist. (Å)	Pose/Affinity (kcal/mol)	Group	Dist. (Å)	Pose/Affinity (kcal/mol)	Group	Dist. (Å)	Pose/Affinity (kcal/mol)	Group	Dist. (Å)	Pose/Affinity (kcal/mol)
DHA	N ζ	3.1	2/−7.0	NH ε	3.0	2/−7.0	NH_2_ η^1^	3.6	2/−7.0	N ε^2^	3.1	1/−7.0	NH_2_ η^1^	3.1	1/−7.0
EPA	N ζ	3.3	2/−6.8	NH ε	3.0	2/−6.8	NH_2_ η^1^	3.3	2/−6.8	N ε^2^	3.5	7/−6.7	NH_2_ η^1^	3.0	7/−6.7

**Table 2:** Electrostatic and hydrogen bond interactions between the carboxylate groups of DHA and EPA and the amino acids of the binding site FA4 of HSA, their poses, and affinities in kcal/mol.

Ligand	R410		Y411			S419		T422		
	Group	Distance (Å)	Group	Distance (Å)	Pose/Affinity (kcal/mol)	Group	Distance (Å)	Group	Distance (Å)	Pose/Affinity (kcal/mol)
	Anchor Site One					Anchor Site Two				
DHA	NH_2_ η^2^	3.1	OH	2.8	3/−7.5	OH	4.6	OH	3.0	2/−7.8
EPA	NH_2_ η^2^	3.2	OH	3.0	3/−7.0	OH	4.5	OH	3.9	2/−7.8

**Table 3:** Electrostatic and hydrogen bond interactions between the carboxylate groups of DHA and EPA and the amino acids of the FA3 of HSA, their molecular docking poses, and affinities in kcal/mol.

Ligands	S342		R348		R485		
	Group	Distance (Å)	Group	Distance (Å)	Group	Distance (Å)	Pose/Affinity (kcal/mol)
DHA	OH	3.0	NH_2_ η^2^	3.0	NH ε	3.1	1/−8.3
EPA	OH	2.7	NH_2_ η^2^	3.0	NH ε	3.0	1/−7.9

**Table 4:** Electrostatic and hydrogen bond interactions between the carboxylate groups of DHA and EPA and the amino acids of the binding site FA6 of HSA, the poses that give rise to the interactions, and affinities in kcal/mol.

Ligands	R209		
	Group	Distance (Å)	Pose/Affinity (kcal/mol)
DHA	NH ε	3.1	5/−7.0
EPA	NH_2_ η^1^	3.1	6/−6.9

## Data Availability

All the data are provided within the article are available from the corresponding authors.

## Supplementary Materials

The following supporting information can be downloaded at: https://www.mdpi.com/article/10.3390/molecules28093724/s1, Figure S1: (A) Selected regions of 2D Tr-NOESY NMR spectra for the binary system DHA (2.5 mM) with warfarin (2.5 mM) in 50 mM PBS buffer in D_2_O with 10% DMSO-*d*_6_. (B) The same solution as in (A) after the addition of HSA (25 μΜ). In both cases, mixing time = 300 ms, T = 310 K, number of scans = 56, experimental time = 15 h. The green cross-peaks in (A) denote intramolecular NOE connectivities of warfarin that are anti-phase with respect to the diagonal. The blue cross-peaks in (B) denote NOE connectivities that are in-phase relative to the diagonal. Figure S2: (A) Selected regions of 2D Tr-NOESY NMR spectra for the binary system EPA (2.5 mM) with warfarin (2.5 mM) in 50 mM PBS buffer in D_2_O with 10% DMSO-*d*_6_. (B) The same solution as in (A) after the addition of HSA (25 μΜ). In both cases, mixing time = 300 ms, T = 310 K, number of scans = 56, experimental time = 15 h. The green cross-peaks in (A) denote intramolecular NOE connectivities of warfarin that are anti-phase with respect to the diagonal. The blue cross-peaks in (B) denote NOE connectivities that are in-phase relative to the diagonal. Figure S3: Selected regions of interligand 2D Tr-NOESY NMR spectra for EPA (2.5 mM) with HSA (25 μΜ) in 50 mM PBS buffer in D_2_O with 10% DMSO-*d*_6_ after the addition of ibuprofen (IB) (2.5 mM) (mixing time = 300 ms, number of scans = 112, experimental time = 17 h). The red cross-peaks denote the interligand NOE connectivities of H5,9, H6,8 (A), H2 (B), and H10,11,3 (C) of ibuprofen with EPA. Figure S4: (A) Selected regions of 2D Tr-NOESY NMR spectra for the binary system DHA (2.5 mM) with ibuprofen (2.5 mM) in 50 mM PBS buffer in D_2_O with 10% DMSO-*d*_6_. (B) The same solution as in (A) after the addition of HSA (25 μΜ). In both cases, mixing time = 300 ms, T = 310 K, number of scans = 56, experimental time = 15 h. The green cross-peaks in (A), which are anti-phase with respect to the diagonal, denote intramolecular NOEs of ibuprofen. The red cross-peaks in (B) denote interligand NOE connectivities that are in-phase with respect to the diagonal.

## Sample Availability

Samples of the compounds are available from the authors.
