# Supplementary material for: Molecular Basis for the Selectivity of DHA and EPA in Sudlow’s Drug Binding Sites in Human Serum Albumin with the Combined Use of NMR and Docking Calculations
Source: Molecules. 2023 Apr 26;28(9):3724. doi: 10.3390/molecules28093724 (PMC10180286; doi:10.3390/molecules28093724)
Supplement: Supplementary file 1 [file molecules-28-03724-s001.zip › molecules-2340805-supplementary.pdf]

SUPPLEMENTARY MATERIAL

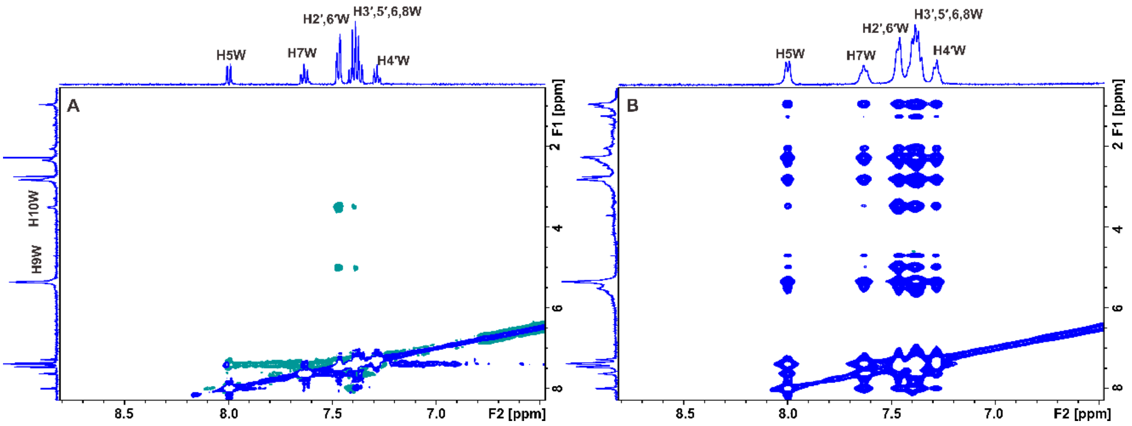

Figure S1. (A) Selected regions of 2D Tr-NOESY NMR spectra of the binary system DHA (2.5 mM) with warfarin (2.5 mM) in 50 mM PBS buffer in D_2O with 10% DMSO-*d*$_6$. (B) The same solution as in (A) after the addition of HSA (25 μM). In both cases mixing time = 300 ms, T = 310 K, number of scans = 56, experimental time = 15 h. The green cross-peaks in (A) denote intramolecular NOE connectivities of warfarin which are anti-phase with respect to the diagonal. The blue cross-peaks in (B) denote NOE connectivities which are in-phase relative to the diagonal.

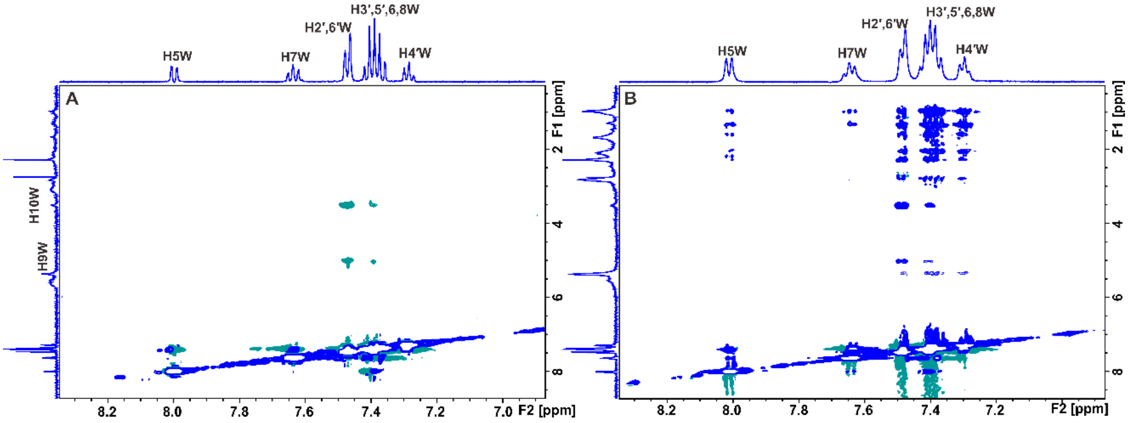

Figure S2. (A) Selected regions of 2D Tr-NOESY NMR spectra of the binary system EPA (2.5 mM) with warfarin (2.5 mM) in 50 mM PBS buffer in D_2O with 10% DMSO-*d*$_6$. (B) The same solution as in (A) after the addition of HSA (25 μM). In both cases mixing time = 300 ms, T = 310 K, number of scans = 56, experimental time = 15 h. The green cross-peaks in (A) denote intramolecular NOE connectivities of warfarin which are anti-phase with respect to the diagonal. The blue cross-peaks in (B) denote NOE connectivities which are in-phase relative to the diagonal.

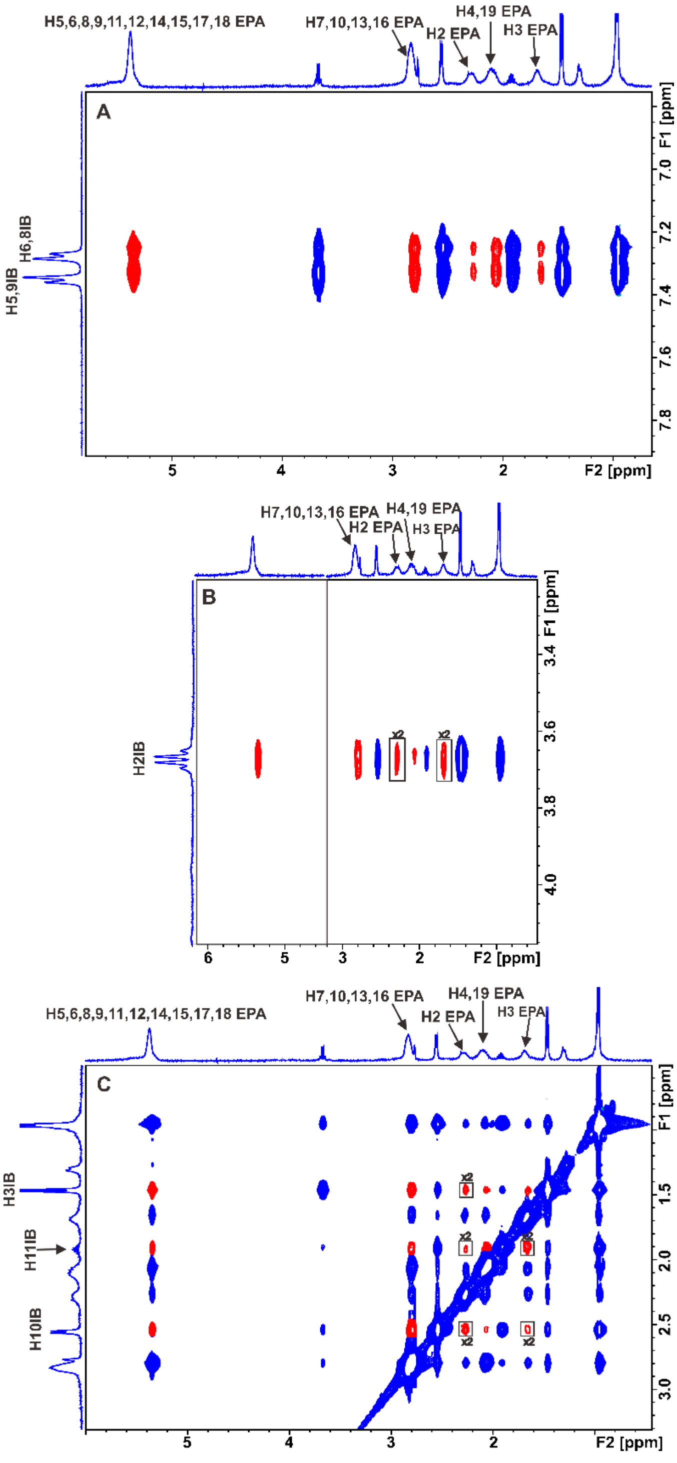

Figure S3. Selected regions of inter-ligand 2D Tr-NOESY NMR spectra of EPA (2.5 mM) with HSA (25 μM) in 50 mM PBS buffer in D_2O with 10% DMSO-d_6 after the addition of ibuprofen (IB) (2.5 mM) (mixing time = 300 ms, number of scans = 112, experimental time = 17 h). The red cross-peaks denote inter-ligand NOEs connectivities of H5,9, H6,8 (A), H2(B) and H10,11,3 (C) of ibuprofen with EPA.

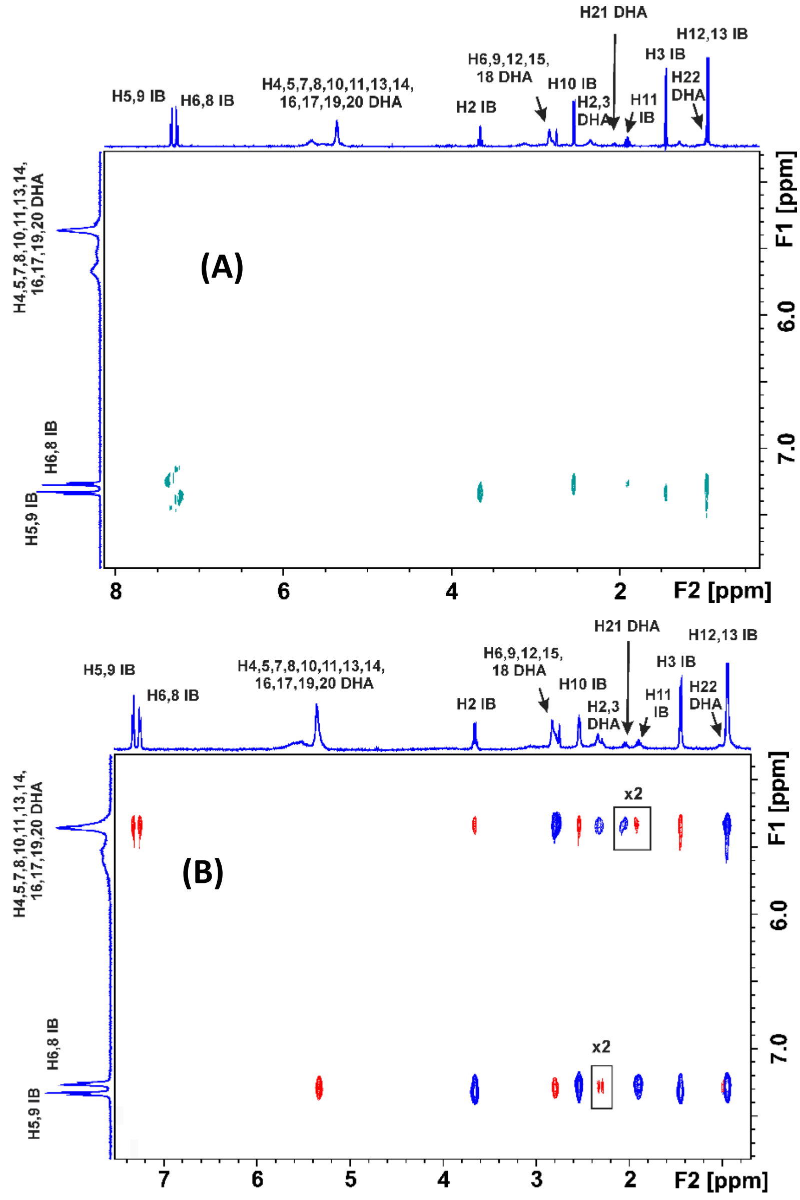

Figure S4. (A) Selected regions of 2D Tr-NOESY NMR spectra of the binary system DHA (2.5 mM) with ibuprofen (2.5 mM) in 50 mM PBS buffer in D_2O with 10% DMSO-d_6. (B) The same solution as in (A) after the addition of HSA (25 μM). In both cases mixing time = 300 ms, T = 310 K, number of scans = 56, experimental time = 15 h. The green cross-peaks in (A), which are anti-phase with respect to the diagonal, denote intramolecular NOEs of ibuprofen. The red cross-peaks in (B) denote inter-ligand NOE connectivities which are in-phase with respect to the diagonal.
